# Ensemble learning-based predictor for driver synonymous mutation with sequence representation

**DOI:** 10.1371/journal.pcbi.1012744

**Published:** 2025-01-06

**Authors:** Chuanmei Bi, Yong Shi, Junfeng Xia, Zhen Liang, Zhiqiang Wu, Kai Xu, Na Cheng

**Affiliations:** 1 School of Biomedical Engineering, Anhui Medical University, Hefei, China; 2 Institutes of Physical Science and Information Technology, Anhui University, Hefei, China; 3 Affiliated Chuzhou hospital of Anhui Medical University, Chuzhou, China; Northumbria University, UNITED KINGDOM OF GREAT BRITAIN AND NORTHERN IRELAND

## Abstract

Synonymous mutations, once considered neutral, are now understood to have significant implications for a variety of diseases, particularly cancer. It is indispensable to identify these driver synonymous mutations in human cancers, yet current methods are constrained by data limitations. In this study, we initially investigate the impact of sequence-based features, including DNA shape, physicochemical properties and one-hot encoding of nucleotides, and deep learning-derived features from pre-trained chemical molecule language models based on BERT. Subsequently, we propose EPEL, an effect predictor for synonymous mutations employing ensemble learning. EPEL combines five tree-based models and optimizes feature selection to enhance predictive accuracy. Notably, the incorporation of DNA shape features and deep learning-derived features from chemical molecule represents a pioneering effect in assessing the impact of synonymous mutations in cancer. Compared to existing state-of-the-art methods, EPEL demonstrates superior performance on the independent test dataset. Furthermore, our analysis reveals a significant correlation between effect scores and patient outcomes across various cancer types. Interestingly, while deep learning methods have shown promise in other fields, their DNA sequence representations do not significantly enhance the identification of driver synonymous mutations in this study. Overall, we anticipate that EPEL will facilitate researchers to more precisely target driver synonymous mutations. EPEL is designed with flexibility, allowing users to retrain the prediction model and generate effect scores for synonymous mutations in human cancers. A user-friendly web server for EPEL is available at http://ahmu.EPEL.bio/.

## Author summary

Although driver synonymous mutations play a crucial role in cancer, their identification is challenged by limited data and intricate pathogenic mechanisms. To overcome these obstacles, we introduced EPEL, a stacking ensemble learning approach for predicting the impact of synonymous mutations in cancer. We systematically explored various novel features, including DNA shape characteristics and chemical molecule-based features. The results show that these features significantly enhance the predictive performance of driver synonymous mutations. We compared EPEL with the other state-of-the-art methods on the independent test dataset. The findings reveal that EPEL substantially enhances the accuracy of identifying driver synonymous mutations. In addition, our findings highlight a critical correlation between effect scores and patient outcomes across various cancer types. It is worth noting that deep biological language models contribute less to the prediction of driver synonymous mutation. We anticipate that these findings will aid in deepening the understanding of driver synonymous mutations.

## Introduction

Due to the degeneracy of codons, where an amino acid can be encoded by multiple synonymous codons, synonymous single nucleotide variants (sSNVs) enable a single base change without altering the encoded amino acid sequence [1]. Although sSNVs do not alter the primary structure of protein, they can impact processes at multiple levels, including DNA transcription, RNA translation, and protein expression [2]. For example, Bhagavatula et al. [3] observed that nine synonymous mutations in *TP53* result in abnormal splicing. The synonymous mutation c.2586G>C alters miRNA binding to receptor tyrosine kinase, preventing miRNA-mediated suppression of receptor tyrosine kinase expression and promoting the development of papillary thyroid carcinoma [4]. It is impractical to detect driver sSNVs *in vitro* from large-scale sSNVs due to the labor-intensive and time-consuming nature of the process. Therefore, an alternative approach is to use *in silico* methods for preliminarily screening of potential driver sSNVs.

Current tools primarily employ various features, such as conservation, sequence and splicing, to characterize deleterious sSNVs from a mechanistic perspective. These methods include CADD [5], DANN [6], FATHMM-MKL [7], PredictSNP2 [8], PhD-SNP^*g*^ [9], FHTHMM-XF [10], regSNPs-splicing [11], IDSV [12], SilVA [13], DDIG-SN [14], TraP [15], synVep [16], PrDSM [17], usDSM [18], EnDSM [19], and frDSM [20]. Among the predictors listed, evolutionary conservation is perhaps the most frequently employed characteristic, except PredictSNP2, synVep, and PrDSM, the rest incorporate conservation feature in their model construction. Additionally, several tools integrate multiple scores derived from annotation tools to construct models that can predict the effect of sSNVs. For instance, PredictSNP2 combines a weighted confidence score from five functional predictors, including CADD, DANN, FATHMM [21], FunSeq2 [22] and GWAVA [23]. Similarly, PrDSM integrates predictive results from TraP, SilVA and FATHMM-MKL. Experimental results demonstrate that integrating functional scores from prediction tools can improve predictive performance. Tools like usDSM, EnDSM, and frDSM incorporate seven scores, including TraP, SilVA, PhD-SNP^*g*^, FATHMM-MKL, CADD, DANN, and FATHMM-XF, along with biological features to construct the predictive model. Despite their widespread use, these predictors may exhibit biases in identifying driver sSNVs, particularly in the context of human cancer complexities. Specifically for cancer-related sSNVs, CSS [24] encompasses seven descriptors, including conservation, local mutation frequency, distance from gene features, GC content, and sequence uniqueness. CS [25] leverages five descriptors, such as amino acid substitutions, conservation, genomic context, and spectrum. While these methods can predict the effect of all sSNVs in human cancer genome, their ability to fully capture the effects of sSNVs in cancer is limited due to the scarcity of sSNVs in the traininig dataset. To address this, a specialized predictor for sSNVs in cancer, named epSMic [26], has been proposed. It incorporates six descriptors, including conservation, splicing, functional scores, sequence, word embedding, and physicochemical properties. Nonetheless, additional features such as DNA shape and deep learning-derived attributes warrant further exploration for their potential to identify driver sSNVs in cancer.

**Fig 1:**
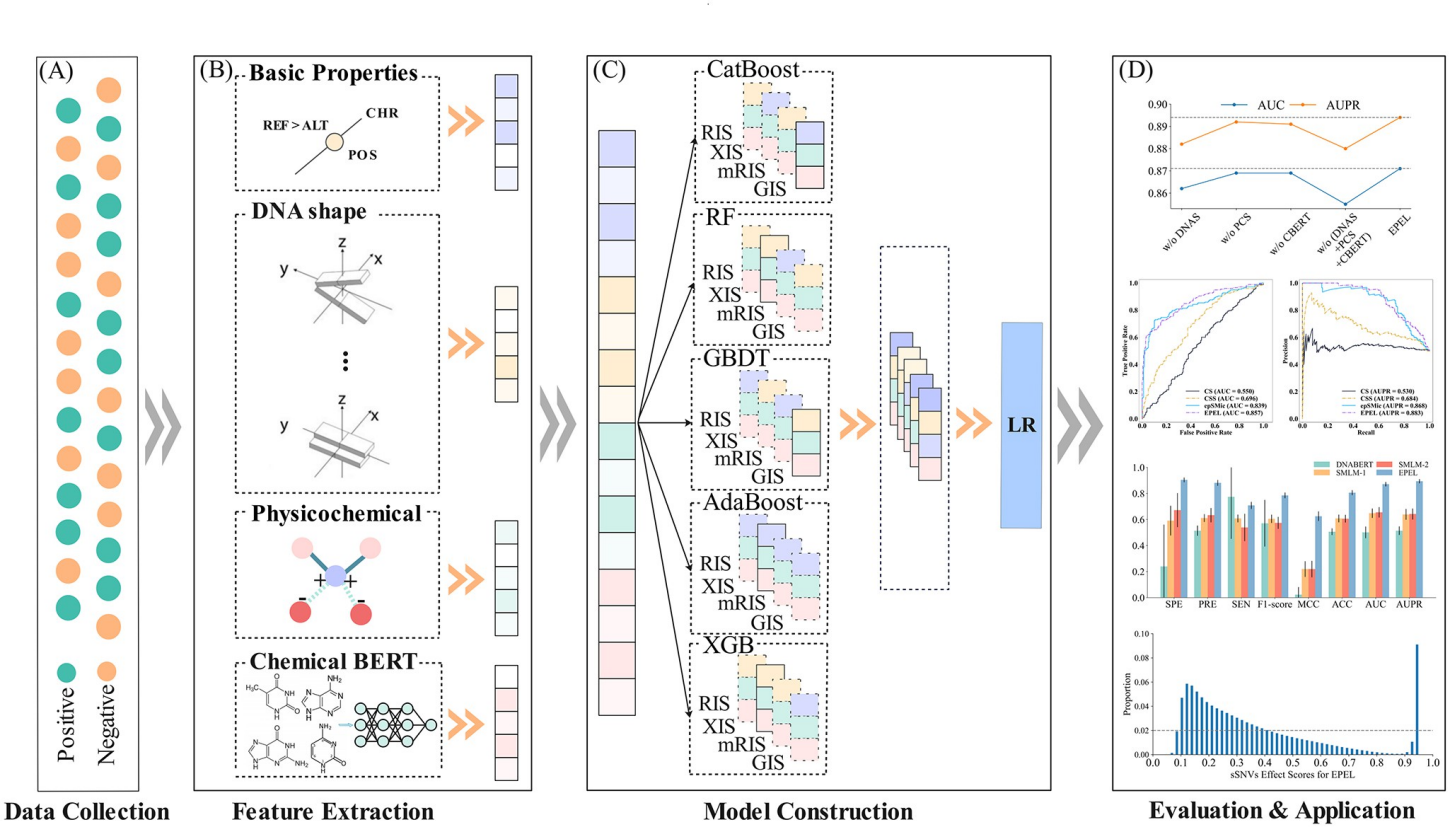
Framework of the proposed EPEL. (A) The curation of training and test datasets. (B) Feature extraction. For a given synonymous mutation, four feature categories of the alternative allele sequences are encoded. Besides, the difference information is also encoded between reference allele sequences and the alternative allele sequences for DNA shape, physicochemical and one-hot encoding of nucleotides, and deep learning-derived features from pre-trained chemical molecular language models. (C) Model construction. Five learners, including CatBoost, RF, GBDT, AdaBoost and XGB, are used to select significant feature subsets and further integrated. Then, feature optimization is performed to obtain less redundant features. Each learner generates probability features based on the corresponding optimal feature selection method with 10-fold cross-validation. Next, generated probability features are further integrated and fed into an LR classifier to generate probability representations as the output of EPEL. (D) Performance evaluation. The performance of EPEL is evaluated through 10-fold cross-validation and independent test dataset. Besides, a web server is also developed for wide users. A score range of 0 to 1 is assigned to sSNVs by EPEL. If the score is > 0.5, it indicates that the synonymous mutation is predicted as a positive sample; otherwise, it is a negative sample.

In this study, we introduced EPEL, an ensemble learning method that utilizes sequence representation to predict driver sSNVs. Initially, we compared seven descriptors, including splicing, functional scores, sequence, conservation, DNA shape, physicochemical properties and one-hot encoding of nucleotides, as well as deep learning-derived features from pre-trained chemical molecule language models. Among these, DNA shape and deep learning-derived features based on chemical molecule were firstly employed for prediction of driver sSNVs. Then crucial feature groups were extracted for modeling. Subsequently, we introduced an ensemble learning method that combines five tree-based models with feature selection methods to distinguish driver sSNVs from passenger ones. The schematic overview of EPEL is shown in Fig 1. Our method demonstrated superior performance compared with other methods on the independent test dataset. Additionally, the results suggest that the effect scores of sSNVs identified by EPEL may correlate with patient outcomes across various cancer types. Ultimately, our findings indicate that despite the success of deep learning methods in other domains, automatic encoding of DNA sequence features through deep learning does not offer significant advantages in this context. We hope that EPEL will accurately identify potential driver sSNVs and facilitate the exploration of their mechanisms in cancer. The web server is available at http://ahmu.EPEL.bio/.

## Results

### The choice of recurrence level

**Fig 2:**
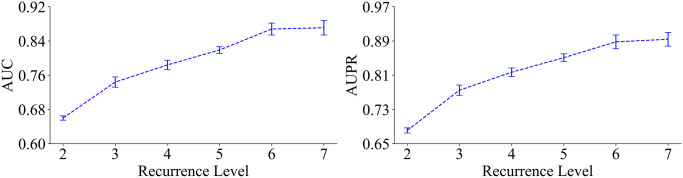
The comparison of thresholds for multiple recurrence levels with 10-fold cross-validation.

Following the previous works [24, 26], we conducted a thorough comparison of different recurrence levels to select suitable positive samples for EPEL. Specifically, sSNVs with recurrence level *r* ≥ k (k = 2, 3, 4, 5, 6, and 7) were considered as putative positive samples, while those with recurrence level *r* = 1 were regarded as putative negative samples. The results for the area under the curve (AUC) and the area under the precision-recall curve (AUPR) are depicted in Fig 2, demonstrating consistent trends across various recurrence thresholds with 10-fold cross-validation. The metrics peak when the recurrence threshold is set to 7 with AUC value of 87.1% and AUPR value of 89.4%. Therefore, we chose *r* ≥ 7 as the positive sample set. Moreover, higher recurrence levels were not considered due to a continuous decrease in sample size (S1 Table) as the recurrence threshold increases. This reduction in sample size may lead to overfitting and diminish the generalization ability of EPEL.

### Assessing the contribution of feature groups

To investigate the contributions of 46 feature groups (see Section *Framework for EPEL* in Materials and methods) in identifying driver sSNVs, we used five tree-based learners, namely categorical boosting (CatBoost), random forest (RF), gradient boosting decision tree (GBDT), adaptive boosting (AdaBoost), and eXtreme gradient boosting (XGB), to evaluate the performance. The results are summarized in S2 Table. In addition to sequence, splicing, and functional scores feature groups, we found that the groups of difference features, including DNA shape, physicochemical properties and one-hot encoding of nucleotides, as well as deep representations features encoded by pre-trained chemical molecule language models, also exhibited positive contributions and achieved AUC and ACC values exceeding 60%. Notably, conservation feature group exhibited weaker performance (AUC = 59.1%). Germline mutations tend to occur in less conservative regions, whereas somatic mutations typically appear in more conserved evolutionary regions [27]. This may explain why the conservation feature group has limited effectiveness in distinguishing driver and passenger somatic sSNVs. Through the feature group selection process, we finally selected 17 feature groups with AUC and ACC values exceeding 60%. This process not only highlights critical features but also reduces feature dimensions and computational complexity.

### Performance comparison of feature selection methods

Four feature importance ranking scores, including RIS, XIS, mRIS and GIS (see Section *Framework for EPEL* in Materials and methods), were applied with five learners to minimize redundancy among feature groups. Specifically, the 134-dimensional features described in Section *Framework for EPEL* in Materials and methods, were ranked in descending order based on importance scores (IS) obtained from RF, XGB, minimal-redundancy-maximal-relevance (mRMR), and GBDT. Subsequently, each of five learners (CatBoost, RF, GBDT, AdaBoost, and XGB) was individually combined with these four IS methods to select the optimal feature subset with 10-fold cross-validation. For each learner, the optimal feature subset was selected to construct a base model. Performance comparison of different feature selection methods is detailed in S3 Table. The results highlight that XIS and GIS consistently outperformed RIS and mRIS across multiple learners. Specifically, CatBoost and GBDT learners identified the same feature subset using GIS, while RF and XGB learners selected the same feature subset using XIS. In contrast, AdaBoost selected the top four features based on XIS. We further investigated the overlap of the optimal feature subsets derived from XIS and GIS, and found that there is a 23.4% overlap in the two selected feature subsets. S1 Fig depicts the optimal feature subset for each learner, emphasizing the critical role of these features in characterizing the impact of sSNVs in cancer.

### The importance for novel features

**Fig 3:**
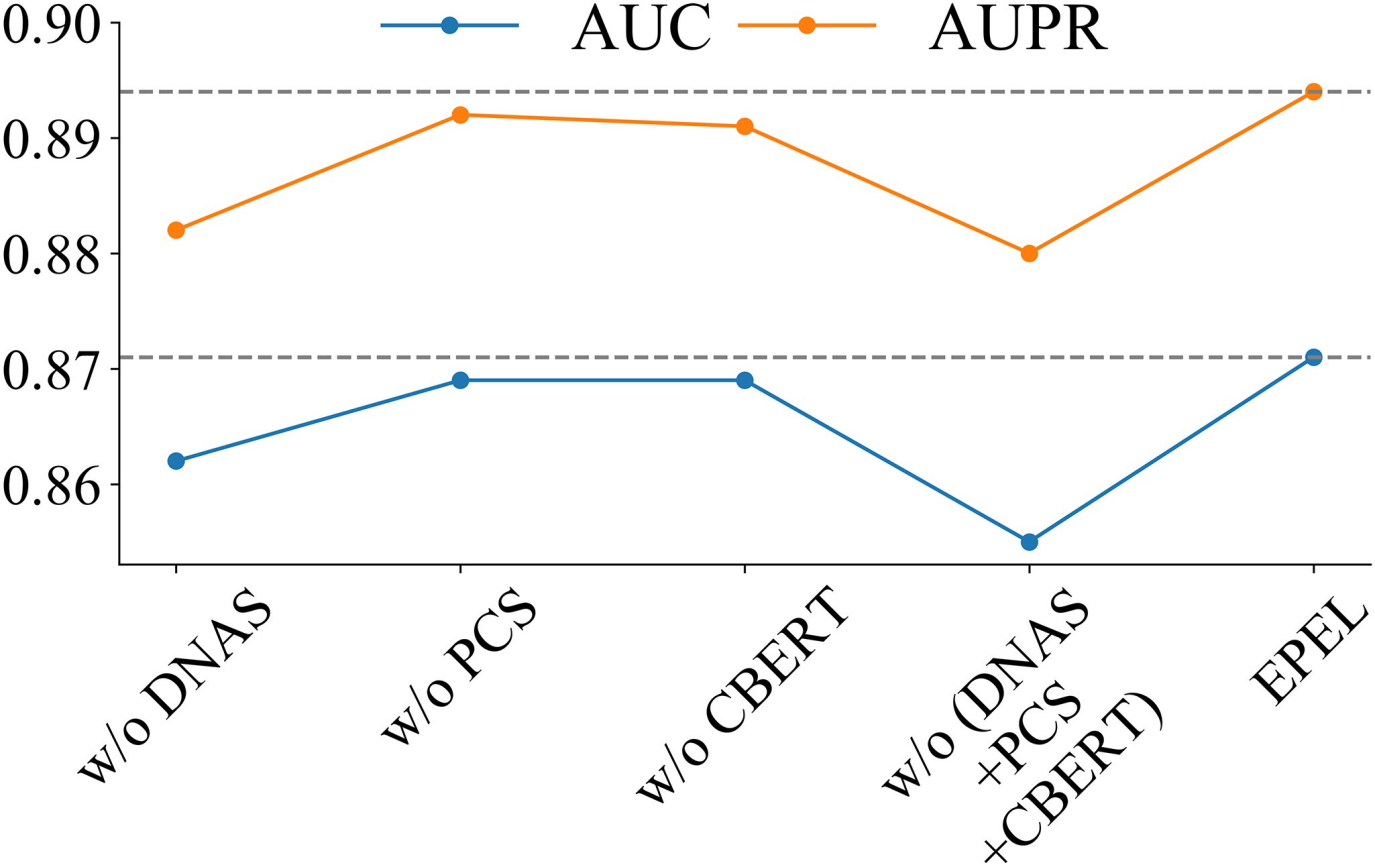
Performance evaluation of the EPEL method with feature sets removed in the training dataset. The difference feature sets include DNAS (difference feature sets of DNA shape between alternative and reference alleles), PCS (difference feature sets of physicochemical properties of nucleotides) and CBERT (difference feature sets of deep representations features generated by pre-trained chemical molecular language models). w/o, without.

To further assess the relative contribution of the novel features employed in EPEL, including DNA shape, physicochemical properties of nucleotides and deep learning-derived features from pre-trained chemical molecular language models, we evaluated the performance by removing the above features and their combinations from the comparison with 10-fold cross-validation. The results are presented in Fig 3. It was observed that the difference feature of physicochemical properties of nucleotides and deep learning-derived features from pre-trained chemical molecular language models make comparable contributions, whereas the difference feature of DNA shape exhibited a greater impact compared to them. Subsequently, we removed the combination of the three feature sets, resulting in the performance dropping substantially, with a 1.6% decrease in AUC and a 1.4% decrease in AUPR, highlighting the positive contribution of these novel features for driver sSNVs prediction.

### Performance comparison of ensemble learning strategies

**Fig 4:**
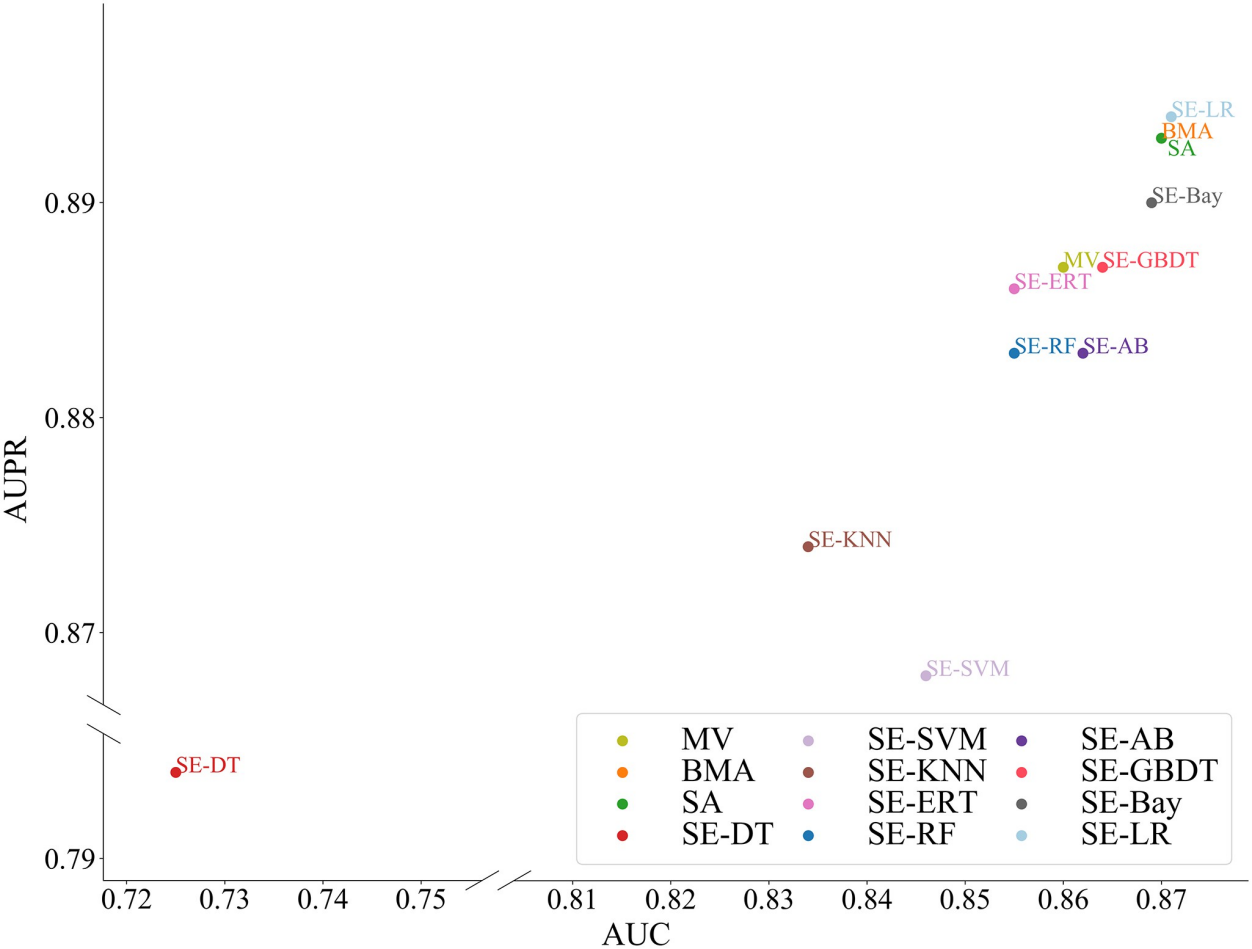
Performance comparison of different ensemble learning strategies with 10-fold cross-validation. SE-LR, EPEL.

Several ensemble learning strategies were employed to build the final model, including majority voting (MV), simple averaging (SA), Bayesian model averaging (BMA) [28], and super-ensemble (SE) [29]. MV is a commonly used ensemble learning strategy, which utilizes the majority predicted labels of various base models as the final output. SA treats each learner equally, averaging their predicted probabilities to generate the final predictions. BMA utilizes a probability density function (PDF) that combines a weighted average of PDFs. In BMA, the weight of each base model reflects its predictive performance relative to others, summing to one and adhering to Bayesian principles. SE, a stacking ensemble learning strategy, constructs final models using predictive probabilities from diverse base models. In this study, we put the predictive probabilities of five base models into nine learners and chose the optimal result to construct the final model, EPEL. The nine learners comprise logistic regression (LR), Support Vector Machine (SVM), RF, Decision Tree (DT), Extremely Randomized Trees (ERT), GBDT, AdaBoost, K-nearest neighbor (KNN) and Bayesian (Bay). Detailed comparison results of these ensemble strategies are presented in Fig 4. It is observed that various ensemble learning strategies exhibit similar performance. Given the robustness and automatic learning capability, we opted for the simple linear super-ensemble, SE-LR, which can automatically learn linear parameters between input features, rather than manually specifying the weights.

### Comparison with cancer-specific predictors

**Fig 5:**
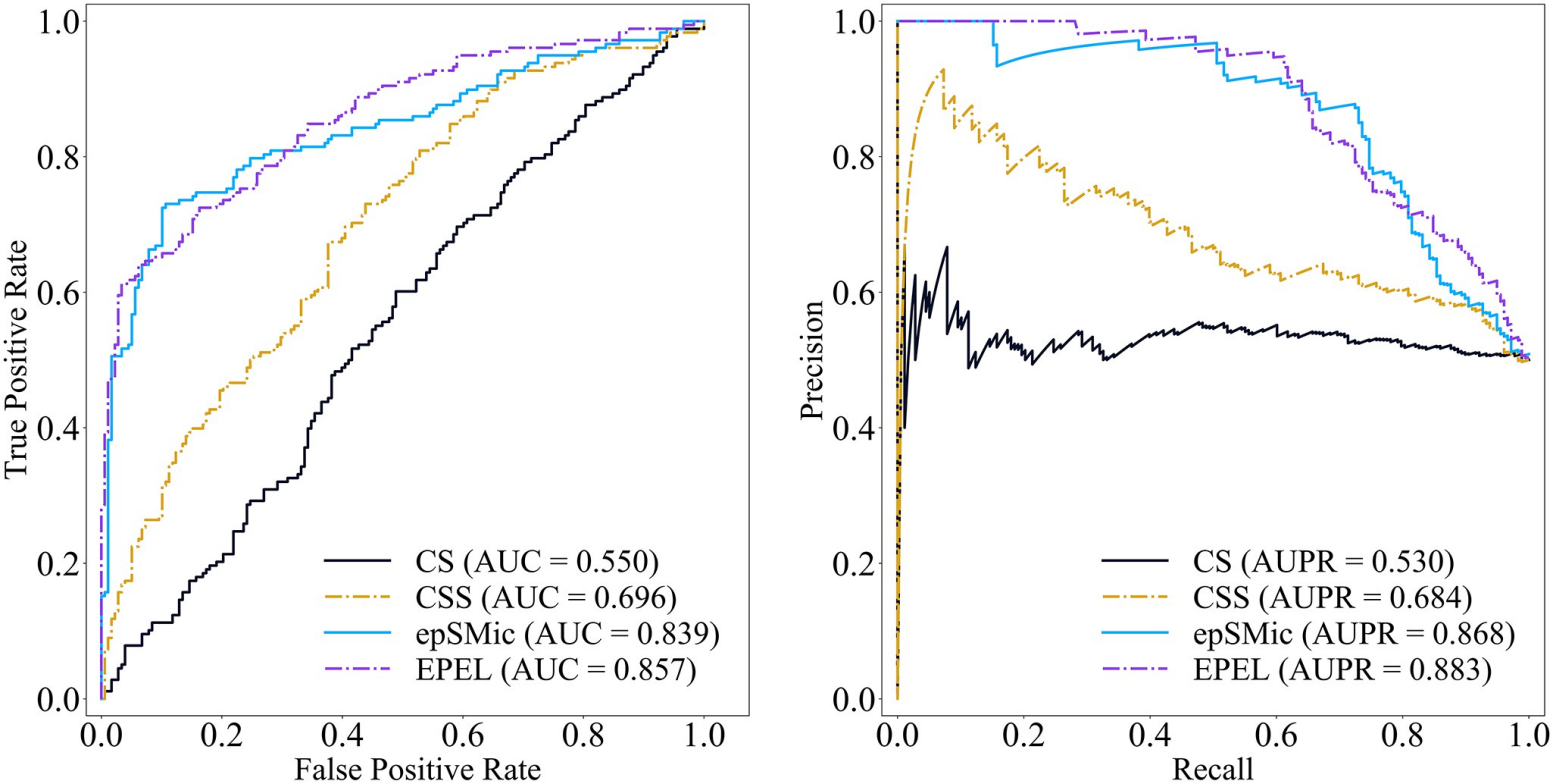
Performance comparison of EPEL with cancer-specific predictors on the consensus independent test dataset.

Based on our previous study [26], we exclusively evaluated EPEL against cancer-specific sSNV methods, including CSS, CS and epSMic on the independent test dataset. Among these, the functional scores for CSS (http://cscape-somatic.biocompute.org.uk/) and CS (http://cscape.biocompute.org.uk/) are derived from their webserver, and the ones for epSMic are obtained from the provided scripts (https://github.com/maxcine-cloud/epSMic). The results (refer to S2 Fig) demonstrated that EPEL outperformed CSS and CS, with an AUC value of 86.2% and AUPR value of 88.4%, while it showed slightly worse than epSMic. In further analysis, we found that 333 sSNVs (317 positive and 16 negative samples) are duplicated with the training dataset of epSMic in our independent test dataset. Thus, we excluded these duplicated sSNVs from independent test dataset to ensure a fair comparison. Given that the missing values (the predictive scores of sSNVs are unavailable) may influence the performance evaluation in comparison with cancer-specific predictors, we then removed missing values (4 positive and 18 negative samples) for CSS and CS from the independent test dataset. Furthermore, we randomly selected 178 passenger sSNVs to match the number of driver sSNVs and named the dataset as consensus independent test dataset. After the process, the consensus independent test dataset includes 178 positive and 178 negative samples. The results (refer to Fig 5) demonstrated that EPEL achieved superior performance, with an AUC of 85.7% and an AUPR of 88.3%. Compared to epSMic, EPEL showed a notable improvement, with a 1.8% increase in AUC and a 1.5% increase in AUPR. As expected, EPEL also outperformed CS by 30.7% and CSS by 16.1% in terms of AUC. It is possible that a small proportion of sSNVs for CS and CSS in the training dataset limited their ability to effectively capture the patterns of driver sSNVs, resulting in inferior performance compared to the driver sSNV-specific prediction tools. Notably, EPEL exhibited higher precision and specificity than epSMic at the default threshold, indicating lower false positives and greater reliability in predicting potential driver sSNVs.

### Interpretation of EPEL scores

**Fig 6:**
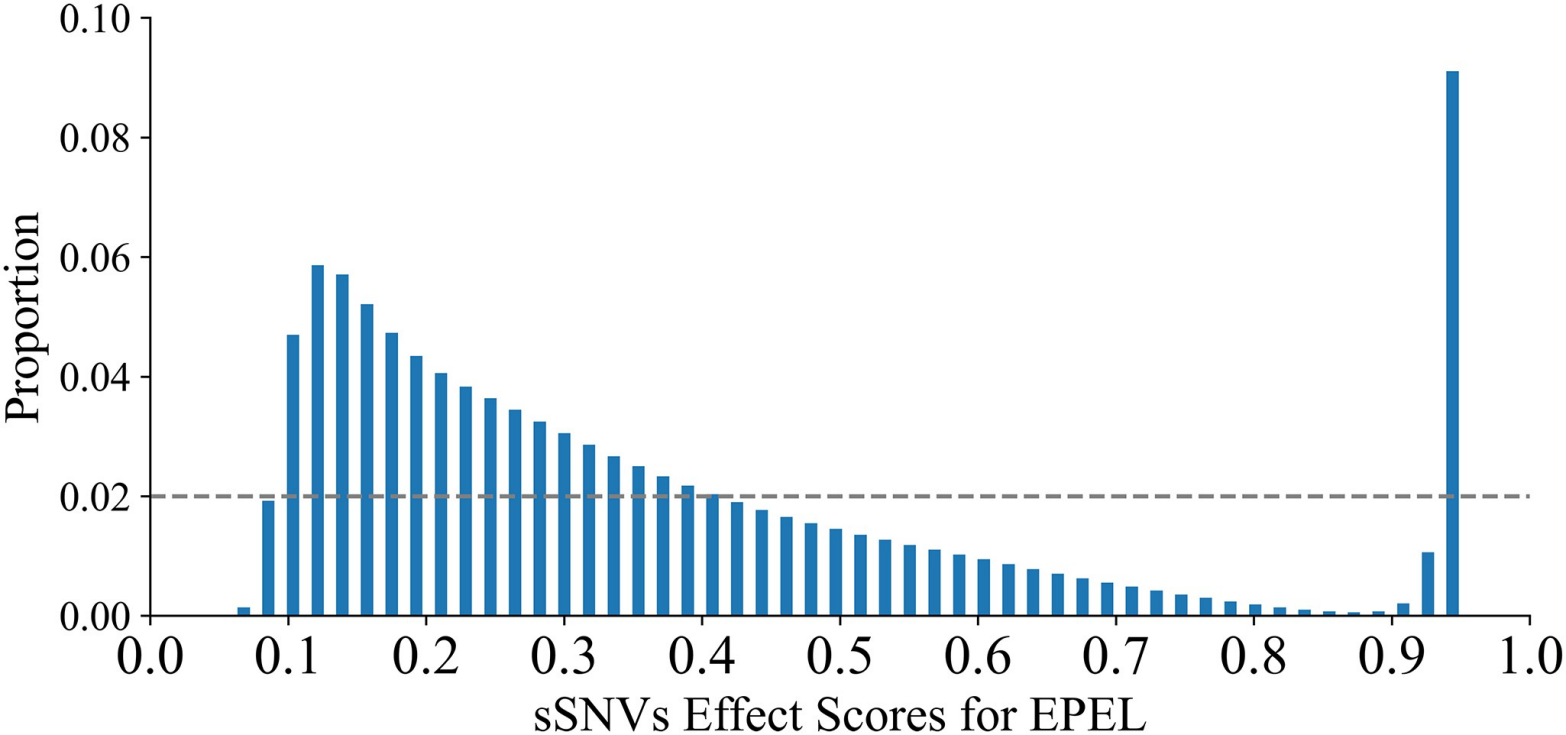
Distribution of EPEL effect scores for all possible synonymous mutations in the human genome. The gray horizontal line denotes the reference uniform distribution.

The EPEL scores, ranging from 0 to 1, reflect the confidence in classifying a synonymous mutation as a driver. EPEL was employed to annotate all potential synonymous mutations in the entire human genome, resulting in the collection of 27,077,126 synonymous mutations to date. The distribution of EPEL effect scores is illustrated in Fig 6, revealing a bimodal pattern across the human genome. The majority of synonymous mutations scored either above 0.9 or below 0.3. Specifically, 53.9% of the synonymous mutations were below 0.3, while 10.4% exceeded 0.9. It is apparent that most driver synonymous mutations were identified with higher confidence across the entire human genome. This demonstrates the capability of the EPEL model to effectively distinguish driver synonymous mutations from passenger ones.

### Analysis the effect of deep biological language model

Deep learning has found widespread application in bioinformatics, such as the identification of pathogenic missense mutations [30, 31]. It automatically learns discriminative features and latent representations of knowledge, reducing the need for manual feature engineering and lowering complexity. To the best of our knowledge, applying automatically encoded deep learning methods to predict the effects of sSNVs has shown limitations [18]. Therefore, our study aims to investigate whether a biological language model can effectively capture the patterns of driver sSNVs.

**Fig 7:**
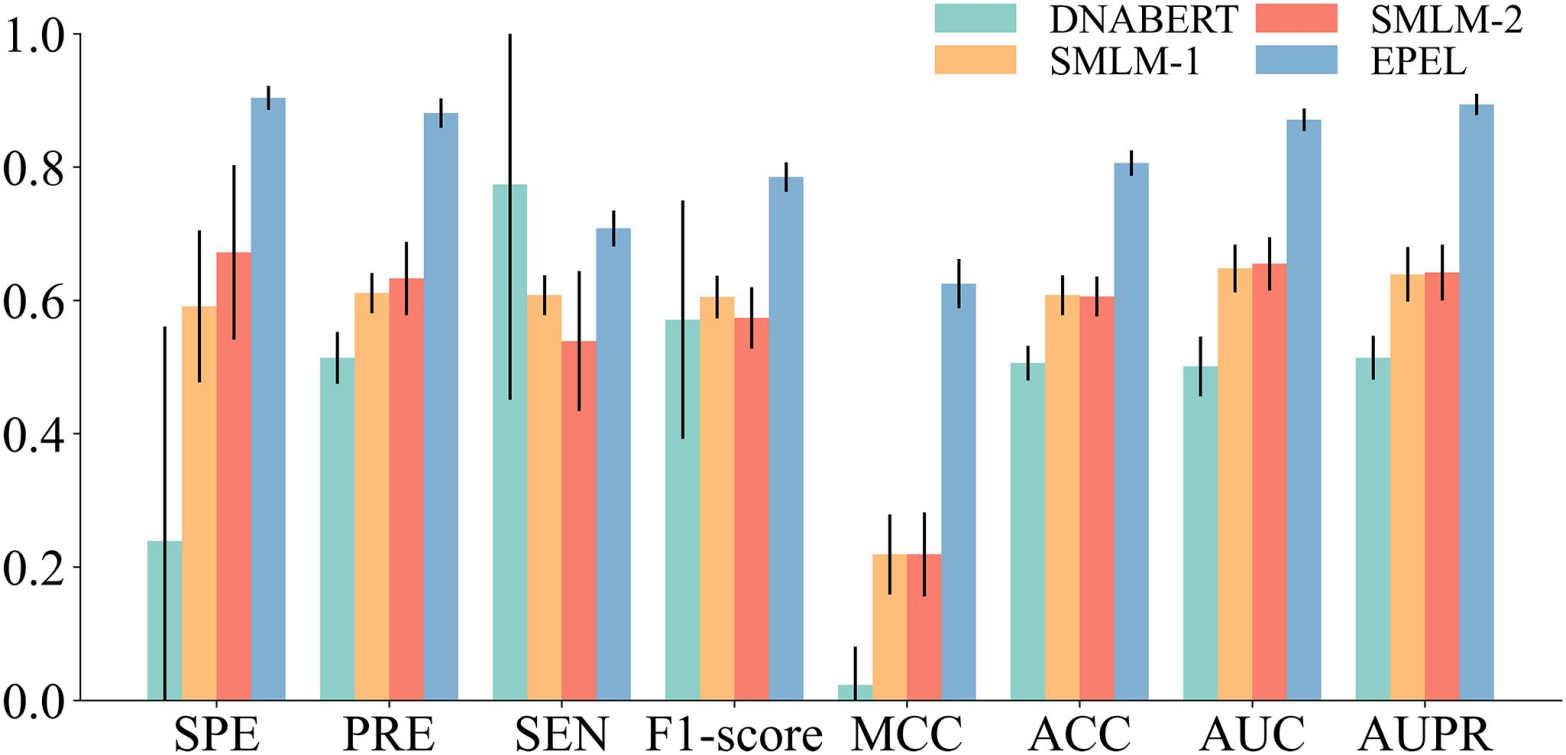
Performance comparison between EPEL and deep biological language models with 10-fold cross-validation.

To represent the effect of sSNVs, we initially developed SMLM-1, a deep biological language model designed to learn DNA sequence patterns. Here, SMLM-1 utilized a multi-head self-attention module from pre-trained DNABERT [32] model. Detailed framework and descriptions of SMLM-1 can be found in S1 Text. To ensure a fair comparison, we employed the same training and test datasets as EPEL. Results revealed that SMLM-1 outperformed DNABERT with a 14.7% improvement in AUC and a 12.5% improvement in AUPR with 10-fold cross-validation (refer to Fig 7). However, EPEL surpassed SMLM-1, achieving a 22.3% improvement in AUC. Considering that the identification of driver sSNVs may rely on prior knowledge used in EPEL, we integrated all features used in EPEL into SMLM-1 through the concatenation module and constructed SMLM-2 model. As shown in Fig 7, SMLM-2 exhibited a 0.7% increase in AUC compared to SMLM-1 but remained sub-optimal compared to EPEL. Possible explanations are as follows. Initially, this disparity may stem from the fact that the representation of driver sSNVs differs from natural language. Furthermore, the small effect sizes of a single synonymous mutation may have limited the capabilities of the deep learning model. Moreover, this observation underscores the complexity of the gene regulatory code involved in identifying driver sSNVs, indicating a need for continued exploration of deep learning methods. Altogether, despite training solely on manually encoded features, EPEL consistently exhibited superior performance compared to several deep biological language learning models.

### Clinical relevance of the cumulative effect risk across cancer types

**Fig 8:**
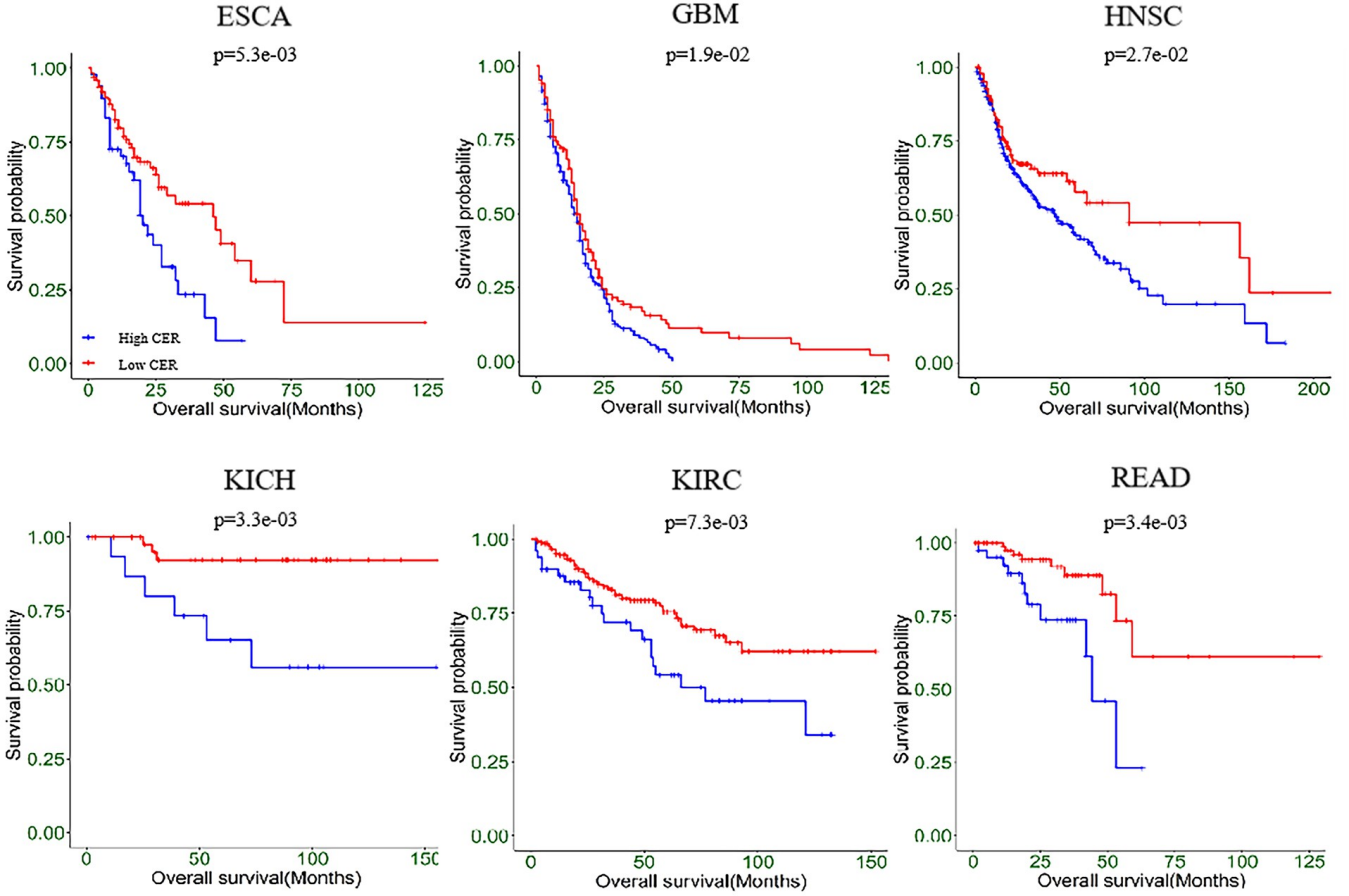
Kaplan-Meier curves of disease-free survival associated with CER values across six cancer types.

To investigate the correlation between effect scores of sSNVs in cancers and patient outcomes, we stratified patients in each cancer type based on cumulative effect risk (CER), which is defined as the sum of scores for driver sSNVs within each patient. Specifically, the somatic sSNVs and clinical data of 33 cancer types (see S2 Text) were collected from UCSC Xena (https://xenabrowser.net/). Subsequently, potential driver sSNVs were identified by EPEL to compute CER values. We use the survcutpoint function from the survminer package in R to determine the optimized CER value, and Kaplan-Meier method and log-rank test to compare the disease-free survival information of patients. We found that there are significant correlations between disease-free survival and CER across 13 cancer types, namely ACC, CHOL, DLBC, ESCA, GBM, HNSC, KICH, KIRC, LGG, MESO, PCPG, PRAD, and READ (see Fig 8 and S4 Fig). Patients with a high CER of driver sSNVs in each cancer type generally exhibited worse survival outcomes compared to those with low CER. Specifically, ESCA, GBM, HNSC, KICH, KIRC and READ have previously shown significant relationships between sSNVs and patient outcomes [33–41], while ACC, CHOL, MESO and PCPG suggest potential influences of sSNVs [42–45]. These findings further revealed the correlation between CER and outcomes of cancer patients, highlighting the potential of CER as an indicator for several cancer types.

### Web server of EPEL

To facilitate the study of potential driver sSNVs, we develop a user-friendly web server, EPEL (http://ahmu.EPEL.bio/). Users can upload a variant call format file or directly input sSNVs into text fields. The required input information includes chromosome, position, reference allele, and altered allele. Users can obtain predictive probabilities within the range [0, 1]. The higher the score, the more likely it is that sSNVs are drivers. Additionally, users can download the pre-computed scores containing all possible sSNVs across the entire genome to facilitate efficient research. Moreover, the EPEL code is available at https://github.com/maxcine-cloud/EPEL, enabling users to retrain the model without any server limitations.

## Discussion

In this study, we proposed an ensemble learning model based on DNA sequence representation to predict the effects of sSNVs in cancer. Initially, we explored the contribution of feature groups, such as DNA shape, physicochemical properties and one-hot encoding of nucleotides, and deep learning-derived features from pre-trained chemical molecular language models. Our findings revealed that the difference features between alternative and reference alleles have significant contribution to model construction. Subsequently, we developed a stacking ensemble learning model based on sequence representation. The results demonstrated that EPEL outperformed other state-of-the-art methods on the independent test dataset, underscoring its effectiveness in identifying driver sSNVs. Furthermore, our analysis of deep biological language models for the identification of driver sSNVs showed their inferior performance compared to EPEL. Additionally, we explored the association between cumulative effect score of sSNVs and cancer patient outcomes. Ultimately, we provided an online web server to facilitate further research. Overall, we hope that effect scores obtained through EPEL are conducive to revealing the mechanisms of driver sSNVs, exploring a significant role in the development and treatment of cancer, and further accelerating drug development and personalized therapy.

However, our research still needs further improvement. Firstly, it is hoped to obtain high-quality data to enhance predictive capabilities, such as experimental or clinical data. Secondly, this study focused on a limited set of features to characterizing sSNVs, such as DNA shape and deep learning-derived features from pre-trained chemical molecular language models, while many other features at various levels, such as genes, transcriptomics, epigenomics and metabolomics, need further investigation. Additionally, cancer type-specific predictive models can be considered for further work to facilitate the development of precision medicine. Lastly, this study exclusively examined the associations between DNA sequence language models and driver sSNVs. Other deep learning techniques warrant exploration, while SMLM-1 may not fully capture the effects of driver sSNVs. For instance, the utilization of variational autoencoders to assess the feature representation capabilities in deep generative models, and the application of meta-learning and unsupervised learning to quickly capture relationships from a handful of samples.

## Materials and methods

### Datasets

The training and test datasets were sourced from the COSMIC database (version 95) [46]. A total of 1,319,113 sSNVs were obtained after removing duplicate samples. Additionally, to avoid data leakage in subsequent clinical relevance, sSNVs that overlapped with the TCGA database were further excluded. Subsequently, redundant sequences of sSNVs were eliminated using the CD-HIT Suite [47] with a threshold of 80%. Following our previous work [26], we utilized the same data construction approach as epSMic, which is based on the recurrence level *r* (the number of synonymous mutations observed in different cases). We hypothesized that highly recurrent sSNVs are more likely to be driver sSNVs, while rare sSNVs are more likely to be passenger sSNVs based on prior observations [24]. Consequently, we defined sSNVs with the recurrence level *r* ≥ 7 as positive samples and those with *r* = 1 as negative samples. Subsequently, we constructed a balanced dataset where each driver synonymous mutation was paired with a closely located passenger synonymous mutation in the genome. The dataset was then divided into training and independent test datasets with a ratio of 8:2, resulting in 3,826 training samples and 998 test samples. Each synonymous mutation was represented by a truncated sequence of 101 base pairs centered at the variant position using BEDTools getfasta program [48] based on corresponding nucleotide with the reference genome (hg19).

### Feature extraction

Besides the basic features such as sequence, conservation, functional scores and splicing, we incorporated novel features into our analysis. These features include DNA shape, physicochemical properties and one-hot encoding of nucleotides, and deep learning-derived features from pre-trained chemical molecule language models based on BERT. Furthermore, we incorporated allele-specific difference features, which consider the differences in features between alternative and reference alleles. For more detailed information of basic features, please refer to epSMic [26]. Other novel features were summarized in S5 Table. All features were centered and normalized within a score range of [0,1] using the Min-Max Normalization method. Missing values of features were imputed with the mean values attributing the observed data. Details of the novel features are described below.

#### DNA shape features

Research has revealed a strong association between DNA shape and position-specific mutation rates in the human genome, offering insights into the structural foundations of nucleotide mutations. [49]. In this study, DNA shape features were annotated from DNAshapeR [50], a total of 14 feature groups. There are six inter-base pair features, including shift, slide, rise, tilt, roll and helix twist (HelT), six intra-base pair features, including shear, stretch, stagger, buckle, propeller twist (ProT) and opening. Additionally, DNAshapeR includes minor groove width (MGW) and electrostatic potential (EP). As a result, we obtained 14 alternative allele feature groups spanning 1,364 dimensions, and 14 difference feature groups between alternative and reference alleles spanning 76 dimensions.

#### Physicochemical properties and one-hot encoding of nucleotides

The four nucleotides (A, T, G, and C) that are present in DNA sequences possess distinct physicochemical properties, such as ring structures and functional groups. We encoded the physicochemical properties of each nucleotide, encompassing nucleotide chemical properties (NCP), electron-ion interaction pseudopotential (EIIP), and physical properties (PCP). These properties have been widely applied in predicting DNA and RNA methylcytosine sites [51, 52]. Specifically, the PCP values were obtained from http://www.basechem.org/, while the EIIP values were retrieved from original research [53], and the NCP scores were sourced from iDNA4mC [54]. Furthermore, to evaluate the effectiveness of sparse encoding, we employed a one-hot sequence representation of nucleotides. In total, we obtained eight feature groups, including four alternative allele feature groups spanning 1,616 dimensions, as well as four difference feature groups between alternative and reference alleles spanning 16 dimensions.

#### Deep learning-derived features with chemical molecule properties

Deep learning-derived features based on chemical molecule have been successfully applied in the fields of computational chemistry and bioinformatics [55–59]. Inspired by the study of Yang et al. [60], we utilized deep learning-derived features from three pre-trained chemical molecule language models based on BERT, namely ChemBERTa [61], XLM-RoBERTa [62], and BERT-base [63], to investigate their potential for the prediction of driver sSNVs. Each nucleotide corresponds to a specific chemical molecular structure, which can be represented as strings using the simplified molecular input-line entry system (SMILES) [64]. These representations are tokenized into substructure information and fed into chemical molecular language model to generate molecular representation information. To mitigate the risk of dimensional catastrophe, we extracted the initial 16 dimensions of deep representation information generated from each chemical molecule language models [60]. As a result, we obtained six deep learning-derived feature groups, encompassing three alternative allele feature groups spanning 4,848 dimensions, and three difference feature groups between alternative and reference alleles spanning 48 dimensions.

### Framework for EPEL

In this study, we introduced EPEL, a novel stacking prediction model, aimed at identifying driver sSNVs in the human genome. The framework of EPEL is depicted in Fig 1. We employed an ensemble learning approach that combines five tree-based learners, including CatBoost, Random Forest (RF), Gradient Boosting Decision Trees (GBDT), AdaBoost, and XGBoost (XGB), chosen for their unique advantages. CatBoost can effectively reduce gradient bias and prediction drift. RF can effectively handle the problem of small samples, high-dimensional feature spaces, and complex data structures. GBDT have the ability to discover nonlinear transformations and can handle skewed variables without the need for transformation. AdaBoost with combining rule of thumbs can easily find high-accuracy classifiers and is less susceptible to overfitting. XGBoost controls the complexity of trees and reduces overfitting by introducing regularization terms in the objective function [65]. We employed these classifiers to construct this model with default parameters through the Scikit-Learn package (version 1.0.2). This combination can minimize model’s instability and improve overall performance. Initially, five tree-based learners (CatBoost, RF, GBDT, AdaBoost, and XGB) were employed in conjunction with the aforementioned 8,007-dimensional (39+1364+76+1616+16+4848+48) features to investigate the contribution of 46 (4+14+14+8+6) feature groups in predicting driver sSNVs. We observed that the novel features, such as DNA shape, physicochemical properties and one-hot encoding of nucleotides, and deep learning-derived features also contribute significantly to identifying driver sSNVs, besides the basic features. In total, we extracted 17 feature groups spanning 134 dimensions, according to the ACC and AUC values, both exceeding 60%. To further enhance computational efficiency and performance, we optimized the extracted features following the idea of forward search strategy (SFS). Specifically, we ranked these features in descending order based on their IS derived from four feature importance measures, including RF, XGB, mRMR, and GBDT. For clarity, we denoted these importance scores as RIS, XIS, mRIS, and GIS, respectively. Subsequently, five learners (CatBoost, RF, GBDT, AdaBoost, and XGB) were employed to obtain an optimal feature subset with 10-fold cross-validation. For each learner, we compared four feature importance measures and selected the optimal combination to construct the base model. Consequently, we built five base models and generated 5-dimensional predictive probability features. Finally, these probability features were fed into a LR classifier, aimed at enhancing the predictive ability of driver sSNVs.

### Evaluation metrics

In this study, we employed various widely used metrics to evaluate the performance of EPEL, including precision (PRE), sensitivity (SEN), specificity (SPE), balanced accuracy (BACC), F1-score, Matthews Correlation Coefficient (MCC) and accuracy (ACC). These metrics are calculated as follows:

$$\begin{array}{c} PRE=\frac{TP}{TP+FP} \end{array}$$ (1)

$$\begin{array}{c} SEN=\frac{TP}{TP+FN} \end{array}$$ (2)

$$\begin{array}{c} SPE=\frac{TN}{TN+FP} \end{array}$$ (3)

$$\begin{array}{c} BACC=\frac{SEN+SPE}{2} \end{array}$$ (4)

$$\begin{array}{c} F1-score=\frac{2TP}{2TP+FP+FN} \end{array}$$ (5)

$$\begin{array}{c} ACC=\frac{TP+TN}{TP+TN+FN+FP} \end{array}$$ (6)

$$\begin{array}{c} MCC=\frac{TP\times TN-FP\times FN}{\sqrt{\left(TP+FN)(TP+FP)(TN+FP)(TN+FN\right)}} \end{array}$$ (7)

Among these, TP and TN represent correctly predicted driver and passenger sSNVs, while FP and FN denote incorrectly predicted ones, respectively. Additionally, AUC (area under the receiver operating characteristic (ROC) curve) and AUPR (area under the precision-recall (PR) curve) are used to assess overall performance.

## Supporting information

S1 Fig Different optimal feature subsets for five learners. Darker colors indicate more selection of features from their respective feature groups. Significant overlap between XIS and GIS highlights the importance of corresponding features within those subsets.
(DOCX)

S2 Fig Performance comparison of EPEL with cancer-specific predictors on the independent test dataset. (DOCX)

S3 Fig Overview of the SMLM-1. Initially, SMLM-1 takes the sequences from the reference allele and alternative allele as input. Position information, alternative allele and reference allele sequences are encoded and fed into basic module based on a biological language model (DNABERT) with 12 self-attention layers and 12 heads. Subsequently, a residual module is incorporated to capture local allelic effects derived from the multi-head self-attention layers. Additionally, a mutation type embedding is introduced and combined with the representation from the last hidden layer of self-attention to further learn global interaction representations. Finally, a classifier with a multilayer perception integrated the local and global level representations for the final prediction.
(DOCX)

S4 Fig Kaplan-Meier curves of disease-free survival associated with CER values across seven cancer types. (DOCX)

S1 Text The framework of SMLM-1. (DOCX)

S2 Text The description of 33 cancer types. (DOCX)

S1 Table Training and test datasets based on different recurrence levels. (DOCX)

S2 Table Performance comparison of multiple feature groups with 10-fold cross-validation. (DOCX)

S3 Table Evaluation on different feature selection methods with 10-fold cross-validation. (DOCX)

S4 Table Performance comparison of different k-mer cases for SMLM-1 with 10-fold cross-validation. (DOCX)

S5 Table Description of the novel features. (DOCX)
