# Supplementary material for: Ensemble learning-based predictor for driver synonymous mutation with sequence representation
Source: PLoS Comput Biol. 2025 Jan 6;21(1):e1012744. doi: 10.1371/journal.pcbi.1012744 (PMC11737855; doi:10.1371/journal.pcbi.1012744)
Supplement: S1 Fig — Darker colors indicate more selection of features from their respective feature groups. Significant overlap between XIS and GIS highlights the importance of corresponding features within those subsets. (DOCX) [file pcbi.1012744.s001.docx]

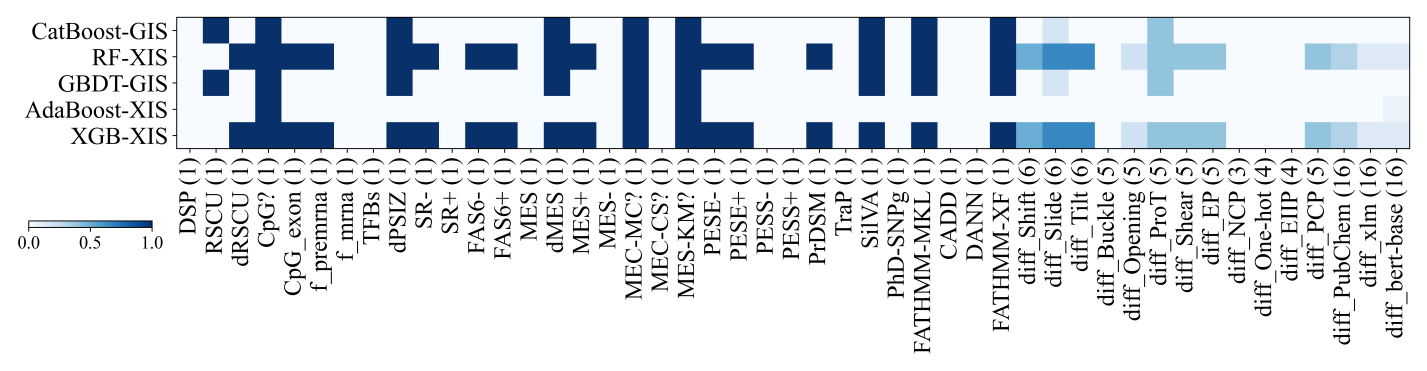


**S1 Fig. Different optimal feature subsets for five learners.**

Darker colors indicate more importance of features from their respective feature groups. Significant overlap between XIS and GIS highlights the importance of corresponding features within those subsets.
