# Supplementary material for: Ensemble learning-based predictor for driver synonymous mutation with sequence representation
Source: PLoS Comput Biol. 2025 Jan 6;21(1):e1012744. doi: 10.1371/journal.pcbi.1012744 (PMC11737855; doi:10.1371/journal.pcbi.1012744)
Supplement: S3 Fig — Initially, SMLM-1 takes the sequences from the reference allele and alternative allele as input. Position information, alternative allele and reference allele sequences are encoded and fed into basic module based on a biological language model (DNABERT) with 12 self-attention layers and 12 heads. Subsequently, a residual module is incorporated to capture local allelic effects derived from the multi-head self-attention layers. Additionally, a mutation type embedding is introduced and combined with the representation from the last hidden layer of self-attention to further learn global interaction representations. Finally, a classifier with a multilayer perception integrated the local and global level representations for the final prediction. (DOCX) [file pcbi.1012744.s003.docx]

**
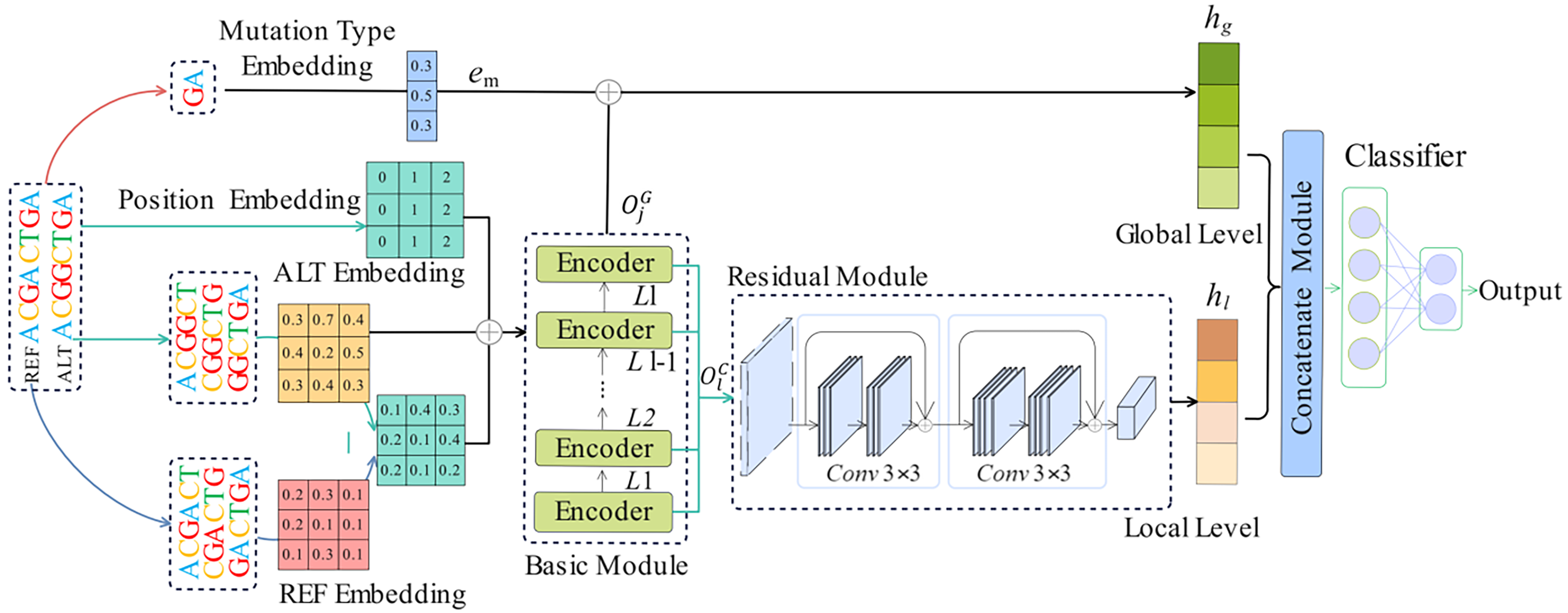
**

**S3 Fig.** **Overview of the SMLM-1.**

Initially, SMLM-1 takes the sequences from the reference allele and alternative allele as input. Position information, alternative allele and reference allele sequences are encoded and fed into basic module based on a biological language model (DNABERT) with 12 self-attention layers and 12 heads. Subsequently, a residual module is incorporated to capture local allelic effects derived from the multi-head self-attention layers. Additionally, a mutation type embedding is introduced and combined with the representation from the last hidden layer of self-attention to further learn global interaction representations. Finally, a classifier with a multilayer perception integrated the local and global level representations for the final prediction.
