# Supplementary material for: Ensemble learning-based predictor for driver synonymous mutation with sequence representation
Source: PLoS Comput Biol. 2025 Jan 6;21(1):e1012744. doi: 10.1371/journal.pcbi.1012744 (PMC11737855; doi:10.1371/journal.pcbi.1012744)
Supplement: S4 Fig — (DOCX) [file pcbi.1012744.s004.docx]

**
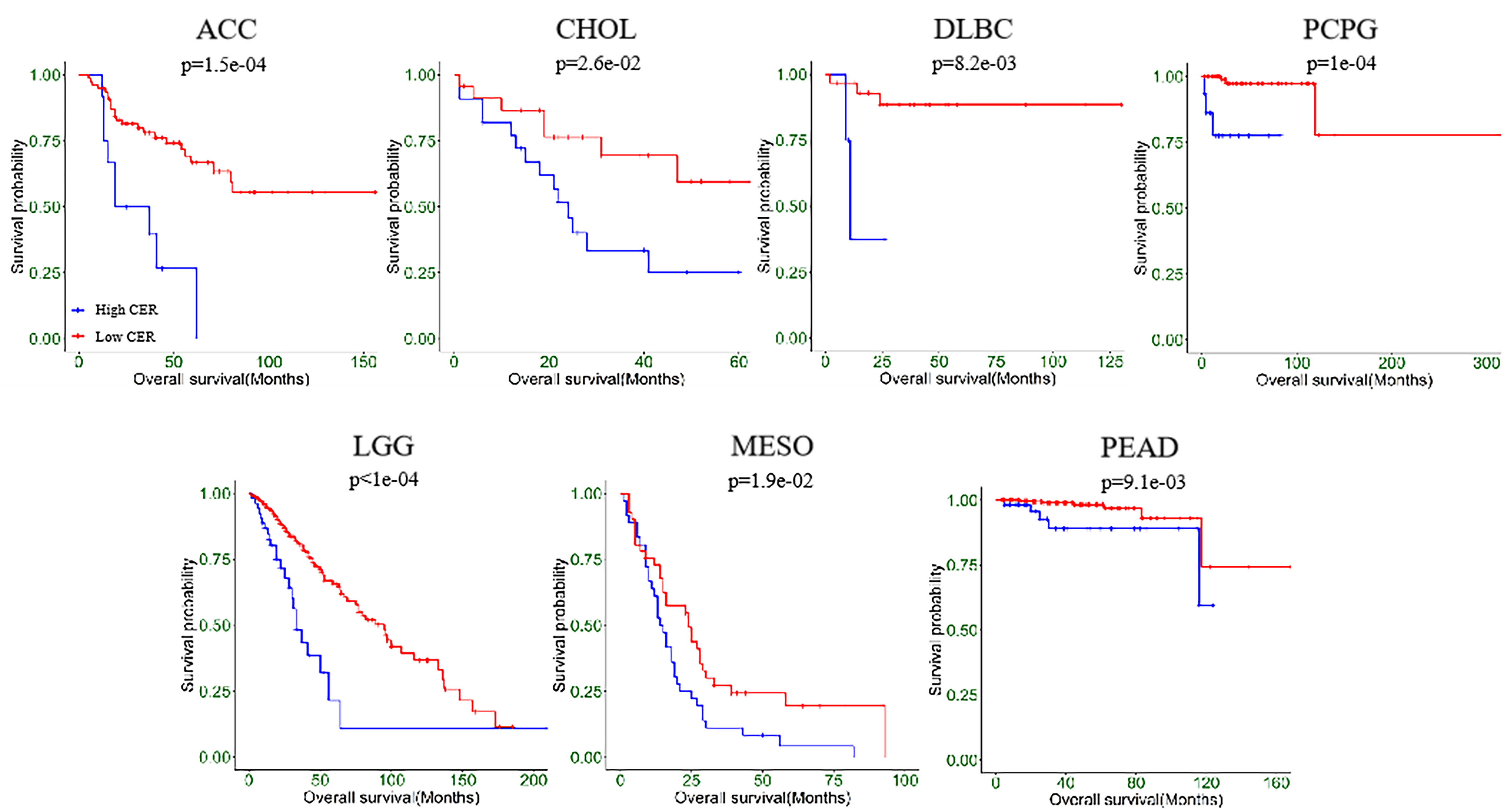
**

**S4 Fig. Kaplan-Meier curves of disease-free survival associated with CER values across seven cancer types.**
