# Supplementary material for: Ensemble learning-based predictor for driver synonymous mutation with sequence representation
Source: PLoS Comput Biol. 2025 Jan 6;21(1):e1012744. doi: 10.1371/journal.pcbi.1012744 (PMC11737855; doi:10.1371/journal.pcbi.1012744)
Supplement: S1 Text — (DOCX) [file pcbi.1012744.s005.docx]

**S1 Text. The framework of SMLM-1.**

SMLM-1 is a deep learning model designed to learn the underlying DNA sequence patterns for predicting the effects of sSNVs in cancer. The architecture is depicted in S3 Fig. Detailed descriptions are provided in the following.

**Sequence representation module**

The sequence representation module converts raw nucleotide sequences into fixed-size numeric feature matrices. Specifically, SMLM-1 processes sequences that include both the reference allele and alternative allele, represented as k-mer tokens. Each token corresponds to *k* bases. We conducted a comparison of four choices of k-mer (*k* = 3, 4, 5 and 6) with a 1-stride setting for genome tokenization. Ultimately, we set *k* = 3 to capture contextual information representation, as it yielded superior performance (see S4 Table). Additionally, five special tokens, namely [PAD], [UNK], [CLS], [SEP], and [MASK], were incorporated. Three embedding layers, comprising alternative allele embedding, position embedding, and differences embedding between the reference allele and alternative allele, were stacked to encode the nucleotide sequences. Formally, a sequence can be represented as *N* tokens, denoted {*t_1_, t_2_, ..., t_N_*}, with *N* represents the token length. The total vocabulary consists of 4*^k^*+5 tokens. For instance, there are 64 traditional token representations and 5 special token representations for *k* = 3. Subsequently, representations from the three layers were aggregated at their respective positions. Each sequence was encoded into a 768-dimensional vector through the embedding module.

An additional sequence representation was incorporated to comprehensively capture the effects of various mutation types. It is important to note that not all token transformations may yield similar outcomes for the effects of sSNVs. Biases in codon usage exist during the translation of the reference genome. Mutation type representations were introduced to simulate the possible sequence transformation following the disruption of codon usage bias. Encoding was performed for 12 mutation types (A>T, A>C, A>G, T>A, T>C, T>G, G>A, G>T, G>C, C>A, C>T, and C>G) to represent their effects. Consequently, each mutation type was converted into a 768-dimensional vector$e_{m}$.

**Basic module with multi-head self-attention**

Here, we employed DNABERT, a pre-trained DNA anguage model based on the BERT architecture. The pre-trained parameters of DNABERT were utilized in SMLM-1, leveraging its ability to capture shared sequence information from the human genome. This transfer not only accelerates convergence with pre-trained parameters, but also allows the model to leverage the common knowledge encoded in DNABERT instead of training from scratch. The basic module consists of multiple multi-head self-attention layers, and the self-attention mechanism of a single head is described as follows.

| $Q_{i}=XW_{i}^{Q}$ | （1） |
| --- | --- |
| $K_{i}=XW_{i}^{K}$ | （2） |
| $V_{i}=XW_{i}^{V}$ | （3） |
| $A_{i}=softmax(\frac{Q_{i}K_{i}^{T}}{\sqrt{d_{k}}})$ | （4） |
| $H_{i}=Attention-Head\left( X \right)=A_{i}V_{i}$ | （5） |

where $Q_{i},K_{i}, V_{i}{\in R}^{N\times d_{k}}$ are query, key, and value matrix respectively, transformed by the output of the sequence embedding module $X$. $\frac{1}{\sqrt{d_{k}}}$ is the scaling factor，${A_{i}\in R}^{N\times N}$represents the attention matrix that determines the inter-token relationships. Then output matrix was generated by applying the scaled dot-product attention weight to the values *V*. To capture more information from diverse perspective, the multi-head self-attention was introduced. The output of multi-head self-attention is as follows.

| $MultiH\left( X \right)=\left[ H_{1},H_{2},\cdots,H_{h} \right]W^{O}$ | （6） |
| --- | --- |
| $O=LayerNorm\left( MultiH\left( x \right)W+X \right)$ | （7） |

where *h* (*h* = 12) represents the number of heads. [·] denotes concatenation across multiple heads. $W^{O}$is a parameter matrix used to map the output dimension of the multi-head self-attention to the initial embedding dimension of the sequence embedding module. Finally, a residual connection and layer normalization operation were introduced to accelerate convergence and better training. The procedure was performed *L* (*L* = 12) times. The basic module passed information with two levels. Global level conveys sequence context information and local level represents changes between alternative allele and reference allele.

**Local module**

To fully capture the information generated by multi-head self-attention, we extracted local representations, $O^{C}=[O_{1}^{C},O_{2}^{C},\cdots,O_{l}^{C}]$, from each layer of the basic module. The local matrix of *i*-th layer, $O_{i}^{C}\in R^{h\times d_{k}}$, measures the average effect associated with mutations tokens (i.e., the average scores of k mutation tokens). Subsequently, the local representation was fed into two residual blocks to encode the integrated local information. Each residual module consists of two convolutional layers, each followed by batch normalization and a *ReLU* activation layer. The two residual blocks have channel sizes of 128 and 256, with a kernel length of 3. Ultimately, these residual blocks are connected to a fully connected layer, which generated the local representation $h_{l}$ for the mutation input sequence segment.

**Global module**

The global representation $O^{G}\in R^{N\times d_{x}}$*,* captures contextual information from all tokens in the final layer. The self-attention mechanism across multiple hidden layers learns contextual information among sequences within the basic module. Subsequently, the global information from the last layer was extracted and combined with the embedded mutation type information to form the output of the global module. The inclusion of embedded mutation type information provides additional guidance for model training and enhances the predictive performance of the model. The global representation can be expressed as follows.

| $h_{g}=W_{3}e_{m}+O_{j}^{G}$ | （8） |
| --- | --- |

**Classification module**

The output *h_g_* from the global module was concatenated with $h_{l}$ from the local module and fed into the classification module to predict the probability of driver sSNVs. Binary cross-entropy loss (Eq (9)) and the Adam optimizer were utilized to train SMLM-1. To prevent overfitting, we also employed mini-batch gradient descent (*N* = 32) and early stopping strategies. SMLM-1 is implemented in PyTorch ([https://pytorch.org/](https://pytorch.org/)).

| $L_{CE}\left( p,y \vert x,\theta\right)=-\frac{1}{N}\sum_{n=1}^{N} ylogp+\left( 1-y \right)\log\left( 1-p \right)$ | （9） |
| --- | --- |

where *p* denotes the prediction probability, *y* represents the true label, *x* is the input, $\theta$indicates the model parameters, and *N* represents the batch size.
