# Supplementary material for: Ensemble learning-based predictor for driver synonymous mutation with sequence representation
Source: PLoS Comput Biol. 2025 Jan 6;21(1):e1012744. doi: 10.1371/journal.pcbi.1012744 (PMC11737855; doi:10.1371/journal.pcbi.1012744)
Supplement: S2 Text — (DOCX) [file pcbi.1012744.s006.docx]

**S2 Text. The description of 33 cancer types.**

In this study, we collected 33 cancers types from were collected from UCSC Xena (https://xenabrowser.net/). According to the primary site, five cancer types related to the genitourinary system, including Bladder Urothelial Carcinoma (BLCA), Adrenocortical Carcinoma (ACC), Kidney Chromophobe (KICH), Kidney Renal Clear Cell Carcinoma (KIRC), and Kidney Renal Papillary Cell Carcinoma (KIRP), seven related to the digestive system, including Colon Adenocarcinoma (COAD), Cholangiocarcinoma (CHOL), Esophageal Carcinoma (ESCA), Liver Hepatocellular Carcinoma (LIHC), Stomach Adenocarcinoma (STAD), Pancreatic Adenocarcinoma (PAAD), and Rectum Adenocarcinoma (READ), two related to the respiratory system, including Lung Adenocarcinoma (LUAD) and Lung Squamous Cell Carcinoma (LUSC), six related to the reproductive system, including Breast Invasive Carcinoma (BRCA), Cervical Squamous Cell Carcinoma and Endocervical Adenocarcinoma (CESC), Prostate Adenocarcinoma (PRAD), Ovarian Serous Cystadenocarcinoma (OV), Uterine Corpus Endometrial Carcinoma (UCEC), Uterine Carcinosarcoma (UCS), and Testicular Germ Cell Tumors (TGCT), three related to the nervous system, including Glioblastoma Multiforme (GBM), Brain Lower Grade Glioma (LGG), and Pheochromocytoma and Paraganglioma (PCPG), three related to the blood and immune system, including Acute Myeloid Leukemia (LAML), Lymphoid Neoplasm Diffuse Large B-cell Lymphoma (DLBC), and Thymoma (THYM), five related to the skin and sense organs, including Skin Cutaneous Melanoma (SKCM), Uveal Melanoma (UVM), Sarcoma (SARC), Mesothelioma (MESO), and Head and Neck Squamous Cell Carcinoma (HNSC), and one related to the endocrine system, including Thyroid Carcinoma (THCA).
