# Supplementary material for: Ensemble learning-based predictor for driver synonymous mutation with sequence representation
Source: PLoS Comput Biol. 2025 Jan 6;21(1):e1012744. doi: 10.1371/journal.pcbi.1012744 (PMC11737855; doi:10.1371/journal.pcbi.1012744)
Supplement: S1 Table — (DOCX) [file pcbi.1012744.s007.docx]

**S1 Table. Training and test datasets based on different recurrence levels.**

| Dataset | Recurrence level *r* | *r*≥2 | *r*≥3 | *r*≥4 | *r*≥5 | *r*≥6 | *r*≥7 |
| --- | --- | --- | --- | --- | --- | --- | --- |
| Training | *pos*. | 61,087 | 21,039 | 10,338 | 5,694 | 3,121 | 1,913 |
|  | *neg.（r=1）* | 61,087 | 21,039 | 10,338 | 5,694 | 3,121 | 1,913 |
| Test | *pos*. | 15,038 | 5,198 | 2,579 | 1,460 | 801 | 499 |
|  | *neg.（r=1）* | 15,038 | 5,198 | 2,579 | 1,460 | 801 | 499 |

The recurrence level of negative samples (*neg*.) is one, and of the positive (*pos*.) samples is under different recurrence levels *r.*
