# Supplementary material for: Ensemble learning-based predictor for driver synonymous mutation with sequence representation
Source: PLoS Comput Biol. 2025 Jan 6;21(1):e1012744. doi: 10.1371/journal.pcbi.1012744 (PMC11737855; doi:10.1371/journal.pcbi.1012744)
Supplement: S2 Table — (DOCX) [file pcbi.1012744.s008.docx]

**S2 Table. Performance comparison of multiple feature groups with 10-fold cross-validation.**

|  | CatBoost | |  | RF | |  | GBDT | |  | AdaBoost | |  | XGB | |
| --- | --- | --- | --- | --- | --- | --- | --- | --- | --- | --- | --- | --- | --- | --- |
|  | ACC | AUC |  | ACC | AUC |  | ACC | AUC |  | ACC | AUC |  | ACC | AUC |
| *sequence (8) | 0.759 | 0.804 |  | 0.782 | 0.826 |  | 0.796 | 0.844 |  | 0.792 | 0.840 |  | 0.765 | 0.820 |
| conservation (7) | 0.557 | 0.584 |  | 0.534 | 0.561 |  | 0.561 | 0.591 |  | 0.548 | 0.570 |  | 0.532 | 0.558 |
| *splicing (16) | 0.789 | 0.811 |  | 0.775 | 0.802 |  | 0.795 | 0.804 |  | 0.794 | 0.803 |  | 0.757 | 0.802 |
| *score (8) | 0.693 | 0.783 |  | 0.752 | 0.814 |  | 0.596 | 0.760 |  | 0.700 | 0.777 |  | 0.644 | 0.706 |
| PCP (505) | 0.595 | 0.626 |  | 0.581 | 0.613 |  | 0.595 | 0.626 |  | 0.569 | 0.599 |  | 0.578 | 0.605 |
| One-hot (404) | 0.591 | 0.624 |  | 0.561 | 0.583 |  | 0.589 | 0.620 |  | 0.577 | 0.608 |  | 0.568 | 0.595 |
| NCP (303) | 0.594 | 0.624 |  | 0.556 | 0.579 |  | 0.580 | 0.619 |  | 0.571 | 0.602 |  | 0.567 | 0.597 |
| EIIP (404) | 0.594 | 0.628 |  | 0.567 | 0.593 |  | 0.589 | 0.620 |  | 0.577 | 0.608 |  | 0.568 | 0.595 |
| *diff_NCP (3) | 0.606 | 0.637 |  | 0.605 | 0.636 |  | 0.606 | 0.637 |  | 0.606 | 0.636 |  | 0.606 | 0.637 |
| *diff_Onehot (4) | 0.606 | 0.637 |  | 0.605 | 0.636 |  | 0.606 | 0.637 |  | 0.606 | 0.616 |  | 0.606 | 0.637 |
| *diff_EIIP (4) | 0.605 | 0.637 |  | 0.605 | 0.636 |  | 0.606 | 0.637 |  | 0.606 | 0.616 |  | 0.606 | 0.637 |
| *diff_PCP (5) | 0.606 | 0.637 |  | 0.605 | 0.636 |  | 0.606 | 0.637 |  | 0.605 | 0.637 |  | 0.606 | 0.637 |
| PubChem (1616) | 0.592 | 0.628 |  | 0.592 | 0.623 |  | 0.583 | 0.618 |  | 0.576 | 0.601 |  | 0.572 | 0.601 |
| xlm (1616) | 0.591 | 0.626 |  | 0.585 | 0.615 |  | 0.588 | 0.617 |  | 0.581 | 0.603 |  | 0.567 | 0.592 |
| bert-base (1616) | 0.596 | 0.626 |  | 0.587 | 0.619 |  | 0.583 | 0.618 |  | 0.576 | 0.601 |  | 0.572 | 0.601 |
| *diff_PubChem (16) | 0.605 | 0.637 |  | 0.605 | 0.636 |  | 0.605 | 0.637 |  | 0.605 | 0.637 |  | 0.605 | 0.637 |
| *diff_xlm (16) | 0.606 | 0.637 |  | 0.605 | 0.636 |  | 0.606 | 0.637 |  | 0.606 | 0.637 |  | 0.606 | 0.637 |
| *diff_bert-base (16) | 0.606 | 0.637 |  | 0.605 | 0.636 |  | 0.605 | 0.637 |  | 0.605 | 0.637 |  | 0.605 | 0.637 |
| HelT (98) | 0.527 | 0.538 |  | 0.516 | 0.528 |  | 0.542 | 0.554 |  | 0.518 | 0.524 |  | 0.514 | 0.523 |
| Rise (98) | 0.550 | 0.568 |  | 0.541 | 0.563 |  | 0.539 | 0.559 |  | 0.529 | 0.540 |  | 0.517 | 0.531 |
| Roll (98) | 0.535 | 0.535 |  | 0.524 | 0.536 |  | 0.538 | 0.543 |  | 0.517 | 0.519 |  | 0.506 | 0.507 |
| Shift (98) | 0.558 | 0.572 |  | 0.541 | 0.552 |  | 0.562 | 0.576 |  | 0.532 | 0.546 |  | 0.535 | 0.547 |
| Slide (98) | 0.555 | 0.578 |  | 0.537 | 0.559 |  | 0.548 | 0.568 |  | 0.535 | 0.543 |  | 0.542 | 0.550 |
| Tilt (98) | 0.560 | 0.581 |  | 0.552 | 0.574 |  | 0.547 | 0.571 |  | 0.546 | 0.561 |  | 0.541 | 0.550 |
| Buckle (97) | 0.560 | 0.585 |  | 0.547 | 0.564 |  | 0.565 | 0.581 |  | 0.551 | 0.564 |  | 0.540 | 0.551 |
| Opening (97) | 0.573 | 0.603 |  | 0.570 | 0.600 |  | 0.581 | 0.605 |  | 0.563 | 0.581 |  | 0.558 | 0.578 |
| ProT (97) | 0.547 | 0.571 |  | 0.546 | 0.564 |  | 0.549 | 0.568 |  | 0.542 | 0.549 |  | 0.545 | 0.558 |
| Shear (97) | 0.597 | 0.632 |  | 0.583 | 0.597 |  | 0.590 | 0.624 |  | 0.582 | 0.613 |  | 0.562 | 0.592 |
| Stagger (97) | 0.530 | 0.549 |  | 0.515 | 0.537 |  | 0.535 | 0.554 |  | 0.526 | 0.545 |  | 0.527 | 0.546 |
| Stretch (97) | 0.533 | 0.548 |  | 0.527 | 0.541 |  | 0.540 | 0.554 |  | 0.523 | 0.537 |  | 0.527 | 0.536 |
| MGW (97) | 0.539 | 0.548 |  | 0.538 | 0.555 |  | 0.527 | 0.538 |  | 0.518 | 0.515 |  | 0.527 | 0.536 |
| EP (97) | 0.582 | 0.604 |  | 0.576 | 0.597 |  | 0.581 | 0.604 |  | 0.555 | 0.579 |  | 0.551 | 0.581 |
| diff_HelT (6) | 0.598 | 0.636 |  | 0.595 | 0.620 |  | 0.599 | 0.634 |  | 0.591 | 0.620 |  | 0.589 | 0.616 |
| diff_Rise (6) | 0.596 | 0.628 |  | 0.575 | 0.606 |  | 0.599 | 0.634 |  | 0.593 | 0.625 |  | 0.568 | 0.596 |
| diff_Roll (6) | 0.593 | 0.628 |  | 0.578 | 0.599 |  | 0.591 | 0.613 |  | 0.575 | 0.596 |  | 0.574 | 0.598 |
| *diff_Shift (6) | 0.610 | 0.642 |  | 0.593 | 0.628 |  | 0.604 | 0.639 |  | 0.596 | 0.630 |  | 0.585 | 0.611 |
| *diff_Slide (6) | 0.601 | 0.640 |  | 0.597 | 0.628 |  | 0.602 | 0.634 |  | 0.590 | 0.619 |  | 0.589 | 0.616 |
| *diff_Tilt (6) | 0.607 | 0.639 |  | 0.595 | 0.626 |  | 0.604 | 0.638 |  | 0.591 | 0.618 |  | 0.581 | 0.609 |
| diff_Buckle (5) | 0.589 | 0.625 |  | 0.590 | 0.621 |  | 0.597 | 0.627 |  | 0.588 | 0.619 |  | 0.587 | 0.612 |
| *diff_Opening (5) | 0.597 | 0.635 |  | 0.589 | 0.619 |  | 0.603 | 0.637 |  | 0.586 | 0.622 |  | 0.587 | 0.604 |
| *diff_ProT (5) | 0.608 | 0.643 |  | 0.594 | 0.622 |  | 0.617 | 0.643 |  | 0.612 | 0.639 |  | 0.585 | 0.609 |
| *diff_Shear (5) | 0.590 | 0.624 |  | 0.582 | 0.601 |  | 0.590 | 0.628 |  | 0.600 | 0.629 |  | 0.579 | 0.606 |
| diff_Stagger (5) | 0.583 | 0.619 |  | 0.582 | 0.604 |  | 0.595 | 0.629 |  | 0.596 | 0.630 |  | 0.567 | 0.594 |
| diff_Stretch (5) | 0.573 | 0.599 |  | 0.553 | 0.567 |  | 0.577 | 0.600 |  | 0.568 | 0.591 |  | 0.564 | 0.591 |
| diff_MGW (5) | 0.574 | 0.597 |  | 0.570 | 0.598 |  | 0.564 | 0.584 |  | 0.561 | 0.580 |  | 0.552 | 0.585 |
| *diff_EP (5) | 0.597 | 0.635 |  | 0.584 | 0.621 |  | 0.601 | 0.644 |  | 0.603 | 0.636 |  | 0.585 | 0.617 |

The “*” denotes selected feature groups. The numeric in the parentheses indicates the dimension of the corresponding feature group*.*
