# Supplementary material for: Ensemble learning-based predictor for driver synonymous mutation with sequence representation
Source: PLoS Comput Biol. 2025 Jan 6;21(1):e1012744. doi: 10.1371/journal.pcbi.1012744 (PMC11737855; doi:10.1371/journal.pcbi.1012744)
Supplement: S4 Table — (DOCX) [file pcbi.1012744.s010.docx]

**S4 Table. Performance comparison of different k-mer cases for SMLM-1 with 10-fold cross-validation.**

|  | CatBoost | |  | RF | |  | GBDT | |  | AdaBoost | |  | XGB | |
| --- | --- | --- | --- | --- | --- | --- | --- | --- | --- | --- | --- | --- | --- | --- |
|  | ACC | AUC |  | ACC | AUC |  | ACC | AUC |  | ACC | AUC |  | ACC | AUC |
| mRIS | 0.803 | 0.862 |  | **0.807** | 0.864 |  | 0.801 | 0.856 |  | 0.787 | 0.846 |  | 0.785 | 0.852 |
| RIS | 0.802 | 0.864 |  | 0.801 | 0.864 |  | 0.797 | 0.857 |  | 0.794 | 0.849 |  | 0.788 | 0.852 |
| GIS | **0.804** | **0.866** |  | 0.806 | 0.866 |  | **0.802** | 0.861 |  | 0.794 | 0.851 |  | 0.790 | 0.854 |
| XIS | 0.803 | 0.865 |  | 0.806 | **0.866** |  | 0.797 | **0.863** |  | **0.795** | **0.855** |  | **0.798** | **0.857** |

The bolded part indicates the maximum value in each evaluation metric*.*
