# Supplementary material for: Ensemble learning-based predictor for driver synonymous mutation with sequence representation
Source: PLoS Comput Biol. 2025 Jan 6;21(1):e1012744. doi: 10.1371/journal.pcbi.1012744 (PMC11737855; doi:10.1371/journal.pcbi.1012744)
Supplement: S5 Table — (DOCX) [file pcbi.1012744.s011.docx]

**S5 Table. Description of the novel features.**

| Feature Name | Feature Description | Source |
| --- | --- | --- |
| *DNA shape features* | the structural features for distinct pentamers |  |
| HelT | helix twist for inter-base pair at each nucleotide position based on Monte Carlo simulations | DNAshapeR [1] |
| Rise | rise for inter-base pair at each nucleotide position based on Monte Carlo simulations |  |
| Roll | roll for inter-base pair at each nucleotide position based on Monte Carlo simulations |  |
| Shift | shift for inter-base pair at each nucleotide position based on Monte Carlo simulations |  |
| Slide | slide for inter-base pair at each nucleotide position based on Monte Carlo simulations |  |
| Tilt | tilt for inter-base pair at each nucleotide position based on Monte Carlo simulations |  |
| Buckle | buckle for intra-base pair at each nucleotide position based on Monte Carlo simulations |  |
| Opening | opening for intra-base pair at each nucleotide position based on Monte Carlo simulations |  |
| ProT | prot for propeller twist for intra-base pair at each nucleotide position based on Monte Carlo simulations |  |
| Shear | shear for intra-base pair at each nucleotide position based on Monte Carlo simulations |  |
| Stagger | stagger for intra-base pair at each nucleotide position based on Monte Carlo simulations |  |
| Stretch | strech for intra-base pair at each nucleotide position based on Monte Carlo simulations |  |
| MGW | minor groove width at each nucleotide position based on Monte Carlo simulations |  |
| EP | electrostatic potential at each nucleotide position based on Monte Carlo simulations |  |
| *Physicochemical properties* | the physicochemical properties of each nucleotide |  |
| PCP | physical properties including the molar refractive index, molar volume, isospecific volume, surface tension and polarizability | http://www.basechem.org/ |
| NCP | nucleotide chemical properties with chemical structure and binding property, including ring structure, functional group and hydrogen bond | iDNA4mC [2] |
| EIIP | electron-ion interaction pseudopotential with the energy of delocalized electrons in amino acids and nucleotides | original research [3] |
| *One-hot encoding* | transforming categorical features for four nucleotides to numerical features | calculation |
| *Deep learning-derived features with chemical molecule properties* | three pre-trained chemical molecule language models based on BERT |  |
| PubChem | large-scale self-supervised pretraining model | ChemBERTa [4] |
| Xlm | unsupervised cross-lingual representation model | XLM-RoBERT [5] |
| Bert-base | pre-training transformers model | BERT [6] |

1. Chiu TP, Comoglio F, Zhou T, Yang L, Paro R, Rohs R. DNAshapeR: an R/Bioconductor package for DNA shape prediction and feature encoding. Bioinformatics. 2016;32(8):1211–1213. doi:10.1093/bioinformatics/btv735.
2. Chen W, Yang H, Feng P, Ding H, Lin H. iDNA4mC: identifying DNA N4-methylcytosine sites based on nucleotide chemical properties. Bioinformatics. 2017;33(22):3518–3523. doi:10.1093/bioinformatics/btx479.
3. Nair AS, Sreenadhan SP. A coding measure scheme employing electron-ion interaction pseudopotential (EIIP). Bioinformation. 2006;1(6):197.
4. Chithrananda S, Grand G, Ramsundar B. ChemBERTa: large-scale self-supervised pretraining for molecular property prediction. arXiv preprint arXiv:201009885. 2020;doi:10.48550/arXiv.2010.09885.
5. Conneau A, Khandelwal K, Goyal N, Chaudhary V, Wenzek G, Guzm´an F, et al. Unsupervised cross-lingual representation learning at scale. arXiv preprint arXiv:191102116. 2019;doi:10.1371/journal.pone.0257230.
6. Kenton JDMWC, Toutanova LK. Bert: Pre-training of deep bidirectional transformers for language understanding. In: Proc. NAACL. vol. 1; 2019. p. 4171–4186.
